# Taurine regulates insulin release from pancreatic beta cell lines

**DOI:** 10.1186/1423-0127-17-S1-S11

**Published:** 2010-08-24

**Authors:** William J L’Amoreaux, Christina Cuttitta, Allison Santora, Jonathan F Blaize, Janto Tachjadi, Abdeslem El Idrissi

**Affiliations:** 1 Department of Biology, College of Staten Island, The City University of New York, 2800 Victory Boulevard, Staten Island, NY 10314, USA; 2 Doctoral Program in Biology – Neuroscience, The Graduate Center, The City University of New York, 365 Fifth Avenue, New York, NY 10016, USA; 3 Advanced Imaging Facility, College of Staten Island, The City University of New York, 2800 Victory Boulevard, Staten Island, NY 10314, USA; 4 Center for Developmental Neuroscience, College of Staten Island, The City University of New York, 2800 Victory Boulevard, Staten Island, NY 10314, USA

## Abstract

**Background:**

Pancreatic β-cells release insulin via an electrogenic response triggered by an increase in plasma glucose concentrations. The critical plasma glucose concentration has been determined to be ~3 mM, at which time both insulin and GABA are released from pancreatic β-cells. Taurine, a β-sulfonic acid, may be transported into cells to balance osmotic pressure. The taurine transporter (TauT) has been described in pancreatic tissue, but the function of taurine in insulin release has not been established. Uptake of taurine by pancreatic β-cells may alter membrane potential and have an effect on ion currents. If taurine uptake does alter β-cell current, it might have an effect on exocytosis of cytoplasmic vesicle. We wished to test the effect of taurine on regulating release of insulin from the pancreatic β-cell.

**Methods:**

Pancreatic β-cell lines Hit-TI5 (Syrian hamster) and Rin-m (rat insulinoma) were used in these studies. Cells were grown to an 80% confluence on uncoated cover glass in RPMI media containing 10% fetal horse serum. The cells were then adapted to a serum-free, glucose free environment for 24 hours. At that time, the cells were treated with either 1 mM glucose, 1 mM taurine, 1 mM glucose + 1 mM taurine, 3 mM glucose, or 3 mM glucose + 1 mM taurine. The cells were examined by confocal microscopy for cytoplasmic levels of insulin.

**Results:**

In both cell lines, 1 mM glucose had no effect on insulin levels and served as a control. Cells starved of glucose had a significant reduction (p<0.001) in the level of insulin, but this level was significantly higher than all other treatments. As expected, the 3 mM glucose treatment resulted in a statistically lower (p<0.001) insulin level than control cells. Interestingly, 1 mM taurine also resulted in a statistically lower level of insulin (p<0.001) compared to controls when either no glucose or 1 mM glucose was present. Cells treated with 1 mM taurine plus 3 mM glucose showed a level of insulin similar to that of 3 mM glucose alone.

**Conclusions:**

Taurine administration can alter the electrogenic response in β-cell lines, leading to a change in calcium homeostasis and a subsequent decrease in intracellular insulin levels. The consequence of these actions could represent a method of increasing plasma insulin levels leading to a decrease in plasma glucose levels.

## Background

The endocrine pancreas is regulated by neurotransmitters, including the use of glutamate and GABA to regulate insulin and glucagon release as well as somatostatin to regulate both α- and β-cell activities. For insulin release, a rise in extracellular glucose to ~3 mM initiates co-release of insulin and GABA [1]. The elevated glucose likely stimulates the release of insulin and GABA from the synapse-like microvesicles (SLMV) through changes in ion currents in β-cells [2,3]. While the insulin is free to move to the circulatory system through the interstitial fluid, GABA binds to GABA_A_ receptors on αcells, causing the hyperpolarization of the α-cells, and inhibiting release of glucagon. Glutamate released from α-cells binds to GluR4 receptors on δ-cells, increasing the release of SST [4]. SST binds either to the SSTR2 receptor on the α-cell or the SSTR1 and/or SSTR5 receptor on the β-cell. SSTR2 activation maintains the GABA-initiated inhibition of glucagon from α-cells, while the SSTR1/5 receptors are responsible for inhibiting insulin release [5].

Taurine (2-aminoethanesulfonic acid) is a sulfur-containing amino acid and is developmentally high in neonates, especially in the brain, and the levels decline to reach stable adult concentrations that are second to those of glutamate in the brain. In the adult, taurine is responsible for maintaining intracellular osmotic balance in a variety of cells examined [6-8]. In the non-obese diabetic mouse model, taurine alters islet development [9].  Previous work done in our laboratory demonstrate that taurine administration during early development in the mouse causes an increase in the number and size of pancreatic islets, without affecting the exocrine portion of the organ [10]. Further, resting plasma glucose levels in these mice were significantly lower than in age-matched controls (manuscript in preparation). When we examined the relative immunoreactivity of insulin, glucagon, and somatostatin in the pancreas of these mice, we found a significant increase in the levels of all three islet markers.

To determine the level at which taurine may interact with the pancreatic β-cells, we set out to test our hypothesis that taurine influences insulin release through its electrogenic transport into the cell. We used immunohistochemistry to address relative intracellular levels of insulin in Hit-T15 (pancreatic β-cell line) and Rin-m (insulinoma) cells.

## Methods

*Cell Culture.* Pancreatic β-cell lines Hit-T15 from Syrian hamster (CRL-1777; ATCC, Manassas VA) or Rin-m (CRL-2057; ATCC) were used for these experiments. Our rationale to use two cell lines arises from the biochemical differences between the two lines. The Hit cells exhibit characteristics of normal β-cells in that insulin production decreases with age and they express receptors for glucagon and somatostatin. The Rin-m cells are pancreatic islet tumor cells and overproduce insulin. All cells were from early passages to prevent passage-dependent decreases of insulin synthesis by the Hit cells. Cells were grown in RPMI medium containing 2 mM L-glutamine, 1.5 g/L sodium bicarbonate, 10% horse serum, 2.5% fetal bovine serum, 50 U penicillin and 50 μg streptomycin (Invitrogen, Carlsbad CA) at 37^o^C, 5% CO_2_. For treatment, cells were plated in complete medium on sterile cover glass at 5,000 cells/cm^2^ until ~80% confluent. The media were removed and replaced with glucose-free RPMI medium that was supplemented with either 1 mM glucose, 1 mM taurine, 3 mM glucose, 1 mM glucose + 1 mM taurine, or 3 mM glucose + 1 mM taurine. *Immunohistochemistry.* Following treatment with the supplemented media, cultures were rinsed in 37^o^C phosphate-buffered saline (PBS) then fixed in 4% paraformaldehyde in PBS. The fixed cells were rinsed in PBS and nonspecific binding blocked with 4% nonfat dry milk, 4% normal goat serum and 0.05% Triton X-100 in PBS. Following blocking, the cultures were incubated with a mouse anti-insulin antibody (Zymed) diluted 1:400 in antibody dilution buffer (1% nonfat dry milk, 2% normal goat serum in PBS). Antibodies were incubated for overnight at 4^o^C with constant agitation. Excess unbound antibody was removed by rinsing in antibody dilution buffer. Following these rinses, the primary antibodies were detected using a goat anti-mouse IgG conjugated with Alexa Fluor 488 (Invitrogen Molecular Probes, Carlsbad CA) or goat anti-mouse IgG conjugated with Alexa Fluor 633 (Invitrogen Molecular Probes, Carlsbad CA). The secondary antibodies were diluted 1: 500 in antibody dilution buffer and cultures incubated for 1 hour at room temperature with constant agitation. Excess unbound secondary antibody was removed by rinses in PBS. The cover glass were placed on a drop of antifade (Slow Fade Gold Plus with DAPI; Invitrogen, Carlsbad CA) and sealed to the microscope slide with nail polish and imaged by confocal microscopy (Leica SP2 AOBS). Laser lines utilized were 405 nm for DAPI, 488 nm for Alexa Fluor, and 633 nm for Cy5. Images were collected using a sequential scan mode to prevent overlap of the DAPI and Alexa Fluor signals. Post-acquisition analyses of relative fluorescence activities were performed by both the Leica software and Imaris X64 (Bitplane Scientific Software, Saint Paul MN).

*Statistical Analyses.* Data from each set of experiments were analyzed by InStat Software (GraphPad Software, La Jolla CA). A one-way ANOVA indicated a significant difference amongst the means (p< 0.001) The Bonferroni multiple comparisons test was then used to compare treatments.

## Results

**Figure 1:**
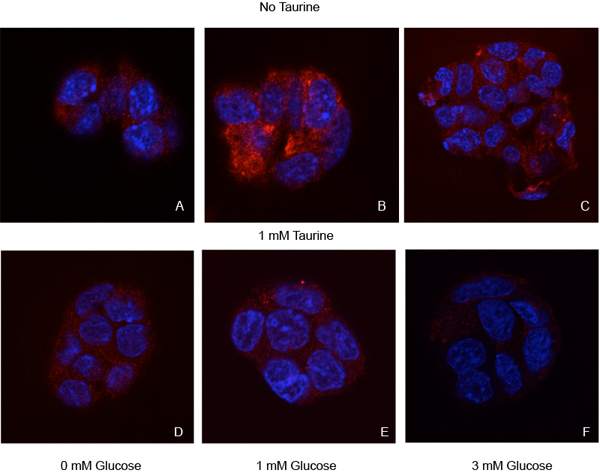
**Insulin immunoreactivity in Hit pancreatic β-cell line.** To test the efficacy of taurine in releasing insulin from the pancreatic β-cell line cultures were treated with no taurine (A-C) or 1 mM taurine (D-F) in increasing glucose concentrations. Cultures received either 0 mM glucose (A, D), the subthreshold concentration 1 mM glucose (B, E), or a concentration shown to initiate glucose release (3 mM; C, F). For controls, we chose the 1 mM glucose without taurine treatment, a condition that does not promote insulin release from β-cells. Cultures were immunostained for insulin (red). Blue structures are nuclei counterstained with DAPI. Insulin levels were significantly decreased in all cultures compared to the control cultures (see Figure 3).

**Figure 2:**
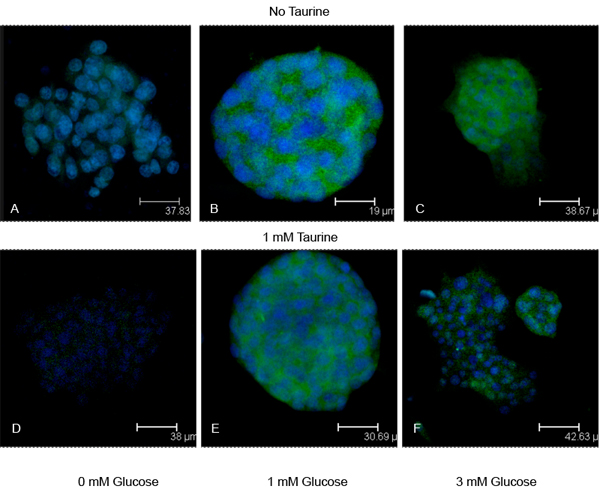
**Insulin immunoreactivity in Rin pancreatic insulinoma cell line.** To test the efficacy of taurine in releasing insulin from the insulinoma cell line cultures were treated with no taurine (A-C) or 1 mM taurine (D-F) in increasing glucose concentrations. Cultures received either 0 mM glucose (A, D), the subthreshold concentration 1 mM glucose (B, E), or a concentration shown to initiate glucose release (3 mM; C, F). Cultures were immunostained for insulin (green). Blue structures are nuclei counterstained with DAPI.

**Figure 3:**
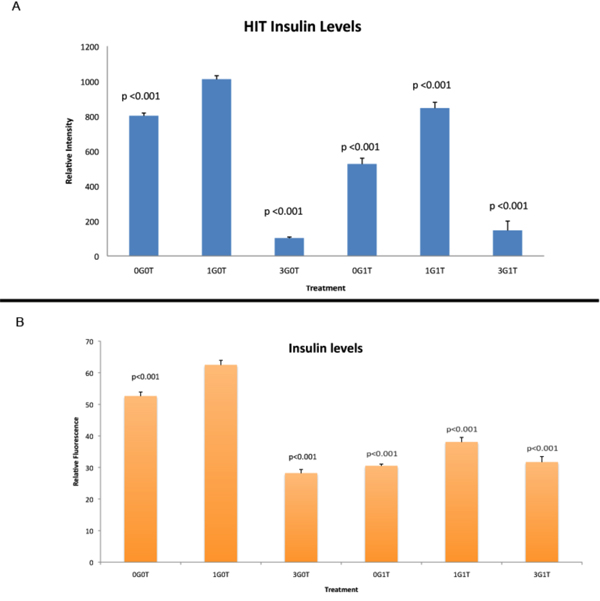
**Comparison of insulin levels in Hit and Rin cells.** Relative fluorescence intensity, a marker for amounts of protein present in the cell, from cells represented in Figures 1 and 2 For statistical analyses, all p values are related to control cultures treated with 1 mM glucose only. A: Relative fluorescence intensity for insulin in the β-cell line Hit-T15. B: Relative fluorescence intensity for insulin in the β-cell line Rin-m. Using 1 mM glucose in the absence of taurine (1G0T) as a control, all treatments resulted in significantly reduced insulin immunoreactivity. Legend: 0G0T (no glucose or taurine), 3G0T (3 mM glucose, no taurine), 0G1T (no glucose, 1 mM taurine), 1G1T (1 mM each glucose plus taurine), 3G1T (3 mM glucose, 1 mM taurine).

Pancreatic β-cells respond to glucose when interstitial fluid levels reach ~3 mM. When we incubate either β-cell line in 1 mM glucose, we find that there are substantial amounts of insulin present in the cytoplasm (Figs. 1b, 2b, 3). Because of the physiological relatedness of this condition, we use the 1 mM glucose as a control for these experiments. When the cells are glucose-starved, there is a significant reduction in the amount of cytoplasmic insulin (Figs. 1a, 2a, 3), likely due to ATP-dependent changes in the K_ATP_-channels. When the cells are treated with 3 mM glucose to approximate *in vivo* conditions, we see a substantial decrease (p<0.001) in insulin immunoreactivity in the cells (Figs. 1c, 2c, 3).

Interestingly, when cells are treated with 1 mM taurine, there is also a significant decrease (p<0.001) in cytoplasmic insulin levels in the cells (Figs., 1d, 2d, 3). In the Hit cells, this decrease is similar to that seen in the glucose-deprived condition (0G0T), with a p value > 0.05 comparing the two treatments. Comparing all other groups, each observed value was statistically different than all others with the exception of the cells treated with 3 mM glucose and 3 mM glucose plus taurine. This suggests that the 3 mM glucose is the overriding stimulus for insulin release.

For the insulinoma cells, again all treatments resulted in a significant decrease in insulin immunoreactivity (p<0.001) compared to controls. As with the Hit cells, insulin immunoreactivity in the presence of 3 mM glucose, while significantly lower than controls, were not significantly different from each other (p>0.05). Of interest, we found that there were no significant differences between Rin cells treated with 3 mM glucose and those treated with 1 mM taurine in the absence of glucose (p>0.05; Fig. 3). Likewise, when taurine is in the presence of glucose, the concentration of glucose does not affect the amount of insulin released as both treatments resulted in no significantly different values for insulin levels (p>0.05). This would seem to indicate that in the insulinoma cell line, either 3 mM glucose or 1 mM taurine could elicit insulin release as effectively as the other.

The Hit cells treated with 1 mM taurine contained more insulin than those treated with 3 mM glucose (Fig. 3), suggesting that different mechanisms exist in normal β-cells than insulinoma cells with regard to sensitivity to taurine-regulated insulin release. When cells were treated with 1 mM glucose (subthreshold) plus 1 mM taurine, both cell lines responded with a significant reduction (p<0.001) in the amount insulin retained compared to controls (Figs. 1e, 2e, 3). In both cell lines, this treatment resulted in significantly higher insulin levels compared to cells treated with 3 mM glucose (p<0.001 for Hit cells; p<0.01 for Rin cells). For cells treated with 3 mM glucose plus 1 mM taurine, the insulin levels were significantly reduced compared to controls (p<0.001; Figs. 1f, 2f, 3). The insulin levels for the synergistic treatment were not significantly higher than the treatment with 3 mM glucose alone (p>0.05).	

## Discussion

We demonstrate here that taurine transport into pancreatic β-cells may initiate the calcium-dependent release of insulin and GABA, independent of extracellular glucose concentrations.  Experimentally, glucose concentrations are considered low if < 2.8 mM but appropriate for insulin release at concentrations above 11 mM, although lower concentrations have elicited physiological responses. Glucose concentrations at 3 mM can regulate glucagon release from pancreatic α-cells, presumably through release of GABA from β-cells [1]. Since insulin and GABA may be co-released, we chose to use 3 mM glucose for our studies. In both cell lines used, this glucose concentration was sufficient to bring about a reduction in intracellular insulin levels compared to concentrations less that 2.8 mM.

Insulin exocytosis begins with the uptake of glucose by the β-cells [2,3] and subsequent breakdown via glycolysis. Subsequent generation of ATP closes ATP-dependent K^+^ channels [11]. Glucose deprivation appears to increase K_ATP_ channel activity through cAMP-activated protein kinase-dependent recruitment of these channel proteins [12]. We demonstrated here that glucose deprivation suppresses insulin release, presumably by cAMP-activated protein kinase-dependent increase in K_ATP_ channel expression at the cell surface [12]. In the paper by Lim et al., the use of 3 mM glucose did not significantly alter K_ATP_-dependent currents; however, in that study the islets were pre-treated with glucose for 2 hr at room temperature. It may be that in our study, the environmental parameters may also contribute to the observed changes in insulin levels. In our study, treatment was for 24 h and at 37^o^C and may more appropriately describe differences seen between our data at 0 mM, 1 mM and 3 mM.

With the significant reduction in insulin using 3 mM glucose compared to 1 mM, we used these two conditions as physiologically relevant. When these cell lines were treated with 1 mM taurine in the absence of glucose, taurine alone was capable of significantly reducing the cytoplasmic insulin levels (Figs 1-3). These data may explain our previous observations that chronic taurine exposure may result in a chronic hypoglycemia. We have found that mice chronically fed taurine in their drinking water are hypoglycemic compared to age-matched controls (data not shown). We further found that when taurine-fed mice are challenged with glucose following a 12 hour fast, there is a delay of 30 min in peak plasma glucose levels, suggesting that these animals are better prepared to handle a sudden rise in glucose. If taurine transport into β-cells increases insulin release, one would expect to find that chronic release of insulin might facilitate glucose uptake, resulting in a chronic depression in plasma glucose concentration.

Additionally, taurine is a known agonist of the GABA_A_ receptor [13-20]. In pancreatic islets, the GABA_A_ receptor is expressed on α-cells where glucagon is synthesized and released. Taurine binding to the GABA_A_ receptor results in a hyperpolarization of α-cells, thus inhibiting glucagon release. For our *in vivo* observations, the hypoglycemia observed in chronic taurine administration may be attributed to activation of insulin release via transport into the β-cells, inhibition of glucagon release via interaction of taurine with the GABA_A_ receptors on the α-cells, or a combination of these events.

## Conclusions

The transport of taurine into pancreatic β-cells may be sufficient to cause the release of insulin. We believe that the mechanism by which this can occur in β-cells involves the interaction of taurine with the ATP-sensitive K-channels. In these cells in vivo, a rise in intracellular glucose levels results in a concomitant increase in cytoplasmic ATP, NADH and NADPH, all required for insulin exocytosis [21]. In part, the mechanism of this action requires an increase in the ATP:ADP ratio, which closes ATP-dependent K^+^ channels [21]. Closure of these K^+^-channels depolarizes the β-cells and activates voltage-gated calcium channels. The resulting increase in cytosolic calcium is responsible in part for the exocytosis of the insulin-containing large dense-core vesicles (LDVC) [21]. The resulting exocytosis of the LDVC also releases GABA, which is found in both the LDVC and synapse-like microvesicles (SLMV) [22]. Therefore, release of insulin and GABA from the pancreatic β-cells requires regulation of both K^+^ and Ca^2+^ currents.

**Figure 4:**
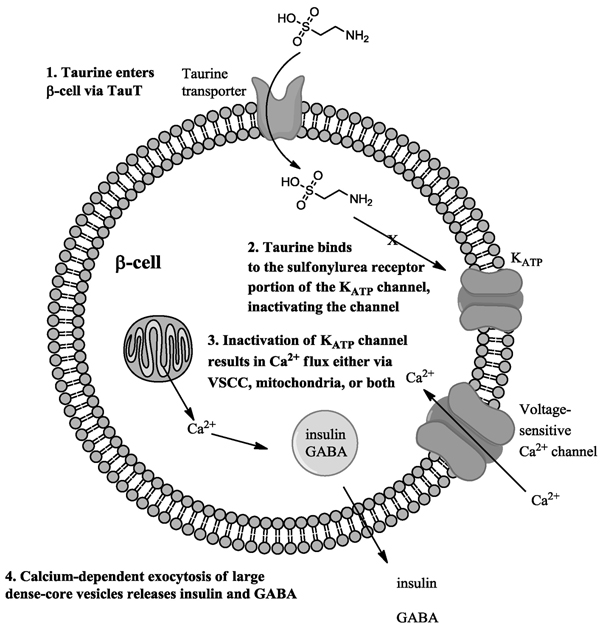
**Proposed scheme by which taurine may affect insulin and GABA release from pancreatic β-cells.** In this scheme, 1: taurine is taken up into the β-cells via the taurine transporter (TauT). Once in the cytoplasm, 2: taurine inactivates the ATP-sensitive K^+^-channels either directly via interaction with the sulfonylurea receptor on the channel, or indirectly via an associated protein that binds to the SUR. The inactivation of ATP-sensitive K^+^-channels leads to 3: an increase in intracellular Ca^++^ either via voltage-sensitive calcium channels or mitochondrial buffering. Once there is a critical Ca^++^ concentration, 4: exocytosis of the large dense-core vesicles containing insulin and GABA occurs. In the in vivo pancreas, GABA would bind to GABA_A_ receptors on α-cells and inactivate glucagon release.

Taurine has been shown to inhibit the K_ATP_-channels in skeletal muscle fibers [23]. In these fibers, taurine binds either to the sulfonylurea receptor (SUR) portion of channel, or to some associated protein that in turn binds to the SUR [23]. We propose the following mechanism by which taurine might independently affect insulin release from the pancreatic β-cells: First, taurine is taken up by the taurine transporter (TauT) on the β-cells (Fig 4, step 1). Once in the cytoplasm, taurine binds to the SUR portion of the channel (or associated protein which in turn binds to the SUR) and inactivates the ATP-sensitive K^+^-channel (Fig 4, step 2). The inhibition of the ATP-sensitive K^+^-channel leads to the opening of voltage-gated calcium channels (Fig 4, step 3) or alternately the efflux of calcium from intracellular stores. In neurons, taurine-modulated calcium flux has been reported [24]. Ultimately, the increase in cytoplasmic calcium concentration results in the exocytotic release of the LDCV containing insulin and GABA.

If taurine is administered chronically, the metabolic relevance could manifest as chronic hypoglycemia. The significance of this in the treatment of type II diabetes could mean that in certain cases, higher plasma insulin levels could alleviate increases in plasma glucose concentrations.

## Competing interests

The authors have no competing interests.

## Authors' contributions

WJL conceived of the study, designed the study, performed the statistical analysis and drafted the manuscript. CC performed the immunohistochemical analyses of the Hit cells; AS performed the immunohistochemical analyses of the Rin cells; JB and JT were responsible for the cell cultures and confocal analyses. AEI participated in the study design and coordination as well as edited the manuscript. All authors read and approved the final manuscript.
